# Lightweight rowers’ perspectives of living with Relative Energy Deficiency in Sport (RED-S)

**DOI:** 10.1371/journal.pone.0265268

**Published:** 2022-03-17

**Authors:** Lucy Gillbanks, Margo Mountjoy, Stephanie R. Filbay

**Affiliations:** 1 Nuffield Department of Orthopaedics, Rheumatology & Musculoskeletal Sciences, University of Oxford, Oxford, United Kingdom; 2 Department of Family Medicine, McMaster University, Hamilton, Canada; 3 International Olympic Committee (IOC)—Games Group, Lausanne, Switzerland; 4 Centre for Health, Exercise and Sports Medicine, Department of Physiotherapy, University of Melbourne, Melbourne, Australia; Yamaguchi University: Yamaguchi Daigaku, JAPAN

## Abstract

**Objective:**

To compete in lightweight rowing, strict limits are placed on the maximum body weight of each individual. As a result, lightweight rowers commonly restrict calorie intake despite high energy expenditure. This can result in Relative Energy Deficiency in Sport (RED-S). The aim of this study is to investigate the physical and psychosocial impact of RED-S, from the perspective of lightweight rowers.

**Design:**

Semi-structured individual qualitative interviews.

**Participants:**

Adults living in the United Kingdom who are current or former lightweight rowing participants and experienced ≥1 symptom of RED-S.

**Method:**

Audio-recorded semi-structured individual telephone interviews were performed. Data was analysed using an inductive thematic approach, coding was iterative and data-driven, facilitated by NVivo software.

**Results:**

Twelve current or former lightweight rowers (intermediate to international standard, 67% female, aged 19–32 years) participated. Participants restricted calories and increased energy expenditure to elicit weight-loss in order to meet weight requirements. This resulted in psychosocial implications (reduced social interaction, difficulty maintaining relationships, poor emotional regulation, low mood, poor concentration, disordered eating, guilt and anxiety around food, and a negative body image). Some psychosocial implications persisted after retirement from lightweight rowing. Participants described a range of physical implications, including disrupted sleep, decreased performance and recovery, bowel disruption, menstrual dysfunction, fatigue, musculoskeletal pain, injury and weakened immune systems.

**Conclusions:**

This study describes short and long-term physical and psychosocial impacts of RED-S from the perspective of lightweight rowers. Findings highlight the importance of effective RED-S prevention and management strategies for lightweight rowers. These findings may be used to educate health-care professionals, coaches and athletes on the personal impacts and serious health consequences of RED-S.

## Introduction

Relative Energy Deficiency in Sport (RED-S) expands upon the concept of the Female Athlete Triad [1]. RED-S recognises that male and female athletes can suffer from the effects of low energy availability, incorporating multi-dimensional symptoms including disruptions in cardiovascular, gastrointestinal, musculoskeletal, haematological, metabolic, endocrine, immunological and psychological function [1]. It is estimated that 70% of elite athletes suffer with ≥1 symptom of RED-S [2, 3]. This high prevalence is of great concern and may impact athletes’ physical and psychological health. An Australian study of elite and pre-elite female athletes found that 80% demonstrated at least one sign of RED-S and one-third of athletes had signs of mental illness [3]. RED-S can go unrecognised and athletes may not receive appropriate education or management [4]. To improve awareness and understanding of RED-S, research exploring the physical and psychosocial impact from the personal perspective of athletes is needed.

Individuals participating in weight-dependent sports, such as lightweight rowing, have a high-risk of developing RED-S due to weight restrictions. Lightweight rowers are required to weigh in 2 hours before racing, with eligibility for summer international competitions requiring men to weigh ≤70kg and women ≤57kg. To achieve weight, athletes use weight loss tactics such as restricting calorie intake, excessive exercise, and increasing sweat loss. Such tactics may increase the risk of developing RED-S symptoms due to increased energy expenditure coupled with low calorie intake [5]. The prevalence and impact of RED-S within this at-risk population is poorly understood and many athletes are unaware of long-term effects [6]. This study aimed to investigate the physical and psychosocial impact of RED-S from the personal perspective of lightweight rowers.

## Methods

This study is reported in accordance with the Consolidated Criteria for Reporting Qualitative Studies (COREQ) guidelines [7] and has been approved by the University of Oxford Medical Sciences Interdivisional Research Ethics Committee (ethics ref: R67265/RE001).

This qualitative study had two overarching aims, however the results for each aim will be reported separately due to depth of responses and manuscript length restrictions. The first aim ‘investigating the physical and psychosocial impact of RED-S from the personal perspective of lightweight rowers’ is addressed in this manuscript. The second aim, ‘investigating knowledge, awareness, and management of RED-S in lightweight rowers and physiotherapists working in lightweight rowing’ is addressed in a subsequent manuscript [4].

A sample of convenience was used to recruit participants via the social media platform Twitter. For participant eligibility, individuals adhered to the following criteria: 1) aged ≥18 years; 2) current or former lightweight rowers from the UK at elite-intermediate level for ≥1 year; 3) self-reported having experienced ≥1 symptom of RED-S whilst participating in lightweight rowing (i.e. recurrent injuries including stress fractures, menstrual dysfunction, low energy levels, prioritised leanness (low body mass taking priority over demands of sport), excessive fatigue, muscle loss, inability to recover between sessions, diagnosis of RED-S or the Female Athlete Triad) [8]. Recruitment posters outlined eligibility criteria and participant eligibility was confirmed via email before a telephone interview was arranged.

Interviews were conducted by one author (LG), a female physiotherapist with four years’ experience competing as an elite lightweight rower. The interviewer had no established relationship with participants prior to interviewing. A semi-structured interview guide was developed in partnership with the target population (lightweight rowers and physiotherapists), and study materials were piloted on two lightweight rowers and two physiotherapists. The semi-structured interview guide contained primarily open-ended questions with relevant prompts to elicit in-depth responses.

One telephone interview per participant was required: duration ranging between 32–60 minutes. A study journal was used, summarising each interview, and allowing reflection of initial thoughts. All interviews were audio-recorded and transcribed verbatim by the interviewer (LG), informed verbal consent was obtained at the start of the interview. Pseudonymisation was used during transcription and an alias was aligned to each participant.

An inductive analysis approach was used; themes were derived from the data, rather than pre-conceptions or theoretical frameworks. Participants’ ‘competition level’ was based on competition category: elite international (top level competition against different countries); elite national (top level competition within the U.K); intermediate (local and national competition racing as an intermediate) [9]. The interviewer (LG) read the transcripts multiple times, with and without the accompanying audio-recording, identifying minor/major themes and similarities/differences between participants’ experiences. NVivo V.11 software facilitated coding of the transcripts into broad-inclusive categories, generating themes/subthemes of relevance to the study aim, whilst refining for analysis. Codes were sorted into hierarchical structure; overlapping codes were merged and codes unrelated to the study aim were filed elsewhere. A second author, experienced in qualitative research (SF) reviewed the data, coding structure and preliminary themes in NVivo V.11 ensuring accurate representation of the dataset, after which a meeting was held to finalise the coding consensus.

## Results

### Participant characteristics

Participants were aged a mean 22 years (range 19 to 32) and 8 (67%) were female (Table 1). Six participants had competed in lightweight rowing at an Olympic or international level (50%), two had competed at a national level (17%), and three had competed at an intermediate level (25%). The length of participation in lightweight rowing competitions ranged from 1 to 8 years. Five participants had retired from rowing completely (42%), four were previous lightweight rowers who were rowing as a heavyweight at the time of interview (33%) and three participants (25%) still competed as lightweight rowers at the time of interview.

**Table 1:** Participant characteristics.

Alias	Age range (years)	Gender	Rowing Status	Competition Level	Lightweight rowing experience
**AMY**	19–21	Female	Former lightweight*	Elite International	1 year
**ANG**	22–24	Female	Former lightweight*	Elite International	2 years
**BOB**	31–33	Male	Former lightweight	Elite International	8 years
**FRED**	19–21	Male	Former lightweight*	Intermediate	2 years
**GRACE**	19–21	Female	Former lightweight	Intermediate	2 years
**MARY**	22–24	Female	Former lightweight	Elite International	5 years
**MEG**	19–21	Female	Current lightweight	Elite National	2 years
**MIA**	19–21	Female	Former lightweight	Elite National	2 years
**PAUL**	19–21	Male	Former lightweight*	Elite International	2 years
**PHILL**	22–24	Male	Current lightweight	Intermediate	5 years
**PIP**	22–24	Female	Current lightweight	Elite national	5 years
**TAL**	22–24	Female	Former lightweight	Elite International	2 years

* Former lightweight rowers who were competing in heavyweight rowing at the time of interview.

### Summary of key themes

**Fig 1:**
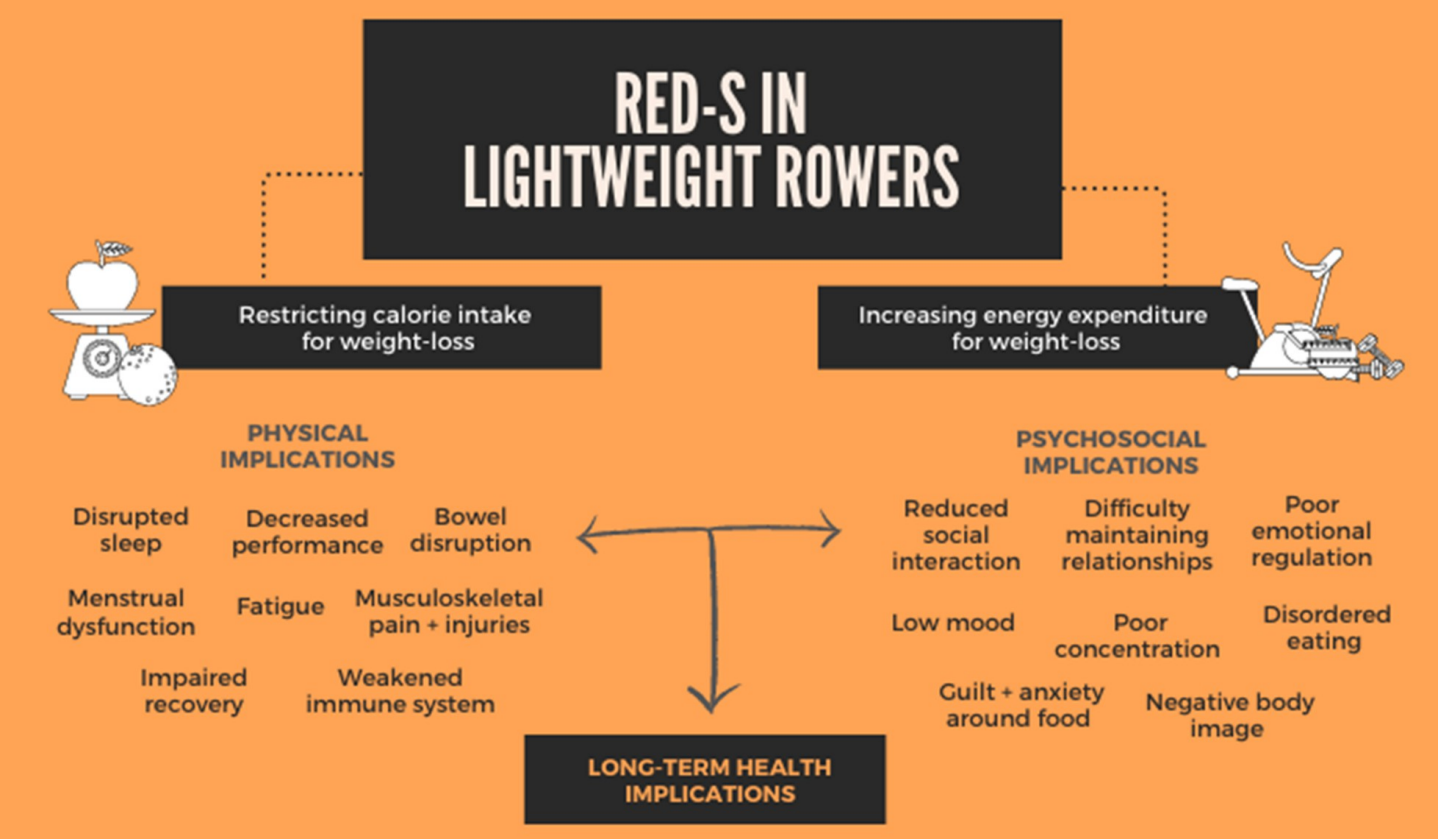
Summary of findings.

All lightweight athletes described restricting calorie intake whilst increasing energy expenditure through excessive exercise and/or weight-loss tactics, prior to weighing in. These practices resulted in a range of physical and psychosocial implications (Fig 1).

### Energy deficiency in lightweight rowers

#### Restricting calorie intake for weight-loss

All athletes described restricting calorie intake to lose weight for lightweight rowing competitions. Weight loss tactics included strict dieting, rituals/routines and fasting in between training.

*BOB*: *‘I kept a training diary for eight years; I would write down every morning and night my weight… I would diet hard*, *teaching myself what the minimum was my body needed to survive on*, *everyone starved themselves*, *there’s days when you literally had no food just like 3 pieces of toast and caffeine in a whole day whilst training 3 times a day*.

*(Age 31–33*, *former lightweight*, *elite international)*

#### Increasing energy expenditure for weight-loss

All athletes increased energy expenditure during weight loss prior to weighing in, involving additional high-intensity cardiovascular exercise on top of 2–3 training sessions each day. Increased energy expenditure was not matched with calorie intake. The mismatch between energy consumption and exertion was seen as desirable and necessary to compete in lightweight rowing. Ten rowers (83%) reported lightweight rowing negatively affected their relationship with exercise and several felt internal pressure to maintain weight with excessive exercise, even once retired.

*AMY*: *‘I’d often go for a walk after dinner*, *to help digestion*. *I’d drink so much water to keep myself full…I would do more cardio based extra exercise… I wouldn’t do lifting weights in gym or if I did it would be like maintenance weight to reduce the weight*, *I would gain* … *my training programme was designed for me to sweat…’*

*(Age 19–21*, former lightweight*, *elite international)*

### Psychosocial implications

Six themes were identified demonstrating the psychosocial implications of RED-S in light-weight rowers, additional quotations are presented for each theme in Table 2.

**Table 2:** Additional quotations illustrating the key themes.

Theme	Participants’ quotes
**The mechanisms of energy deficiency**	
Restricting calorie intake	*ANG*: *‘I used to skip breakfast and do a long session then I’d have like an apple …I’d make myself wait*, *so I was probably just running on empty*.’ *(Age 22–24*, former lightweight*, *elite international)* *PAUL*: *‘…I would always be in a constant cutting state*, *I would never have a way of being able to eat lots of carbs*, *it was only the week after a competition I would be able to have pasta or a curry for dinner*, *otherwise it was just salmon and fish and salad*. *So*, *I never really made a cut because I was always slowly depleting my calories and just constantly cutting to maintain my weight rather than go up and down*.*’ (19–21*, former lightweight*, *elite international)*
Increasing energy expenditure	*A*NG: *‘I was doing a lot more sweaty exercise to help lose the weight*, *whilst feeling awful at the same time but building up to the big competitions we were doing like 3 sessions a day and we would continue to build on this with intensity and length of pieces before tapering just before the race day*.*’* (Age 22–*24*, former lightweight*, *elite international*) *BOB*: *‘110% it [lightweight rowing] affected performance*, *but it was just something you have to do to make weight and race…I hated training because of it*, *I would be training 3 maybe 4 times a day in the peak of summer building up to a race and sometimes eating less than the average person…*.*a lot of training sessions I’d go into feeling pretty knackered… …for a world cup you would have 3 days of racing and need to be 70kg for 3 days in a row*, *sometimes I would need to do extra sweat sessions in between racing or as well as racing to keep the weight off*.*’ (Age 31–33*, *former lightweight*, *elite international)*.
**Psychosocial implications of RED-S**	
Low mood and poor emotional regulation	*ANG*: *‘I struggled in lectures because I was so hungry*. *I got headaches and felt so tired and fatigued*. *I would wake up and feel groggy and horrible… I felt rubbish and just sad*.*’ (Age 22–24*, former lightweight*, *elite international)* *PIP*: *‘Oh building up to a competition every lightweight is very touchy about the subject of weight so if anyone brings up meal time*, *food or appears to be questioning your diet*, *we would just snap… it can often be very tense to be in a lightweight crew… I was completely different when I was dieting to how my usual personality is*.*’*(*A*ge *22–24*, *current lightweight*, *elite national)*
Reduced social interaction and difficulty maintaining relationships	*ANG*: *‘I wouldn’t go out for dinner with friends because I knew I couldn’t eat anything… my mum got the brunt of it… I don’t know how my boyfriend stayed with me*.*’ (Age 22–24*, former lightweight*, *elite international)* *MARY*: *‘I didn’t go to like any of the rowing socials because I knew I would just sit there and not be able to eat anything which would make me more upset than actually going*. *I did have so many incredible friendships built throughout my career but when I was dieting my personality just changed and I wasn’t the same person so it was difficult to maintain those relationships which are built on another type of your personality*.*’ (Age 22–24*, *former lightweight*, *elute international)*
Poor concentration	*PHILL*: *‘My job was based around communication*. *I had less tolerance and concentration levels so I had to try and do things over email just so I didn’t cause drama it gave me a bit more time to think*.*’ (Age 22–24*, *current lightweight*, *intermediate)* *MARY*: *‘In my second year*, *my concentration was definitely worse from being so hungry and thinking of food… I have now split my course year 50% intensity… I was really struggling to make weight*, *cut*, *train*, *along with assignments and exams*.*’ (Age 22–24*, *former lightweight*, *elite international)*
Guilt and anxiety around food	*TAL*: *‘I think it made you feel guilty when you saw some people eating things*. *It made it worth doing with other people though as we were able to help each other through*, *there were times when none of us were eating but we really should have been because of the amount of training we were doing but we were too scared of not making weight and that meant if you saw your teammate struggling or not eating you were like oh I have to do the same*‥ *we were sometimes a kilo underweight because we had gone too hard…and it is a constant stress of feeling guilty and nervous about your next meal and worrying about your weight*.*’ (22–24*, *former lightweight*, *elite international)* *PHILL*: *‘…I wanted someone to tell me not to feel guilty about what I eat because that was the biggest thing for me*, *feeling guilty about food… I definitely skipped meals when I was cutting because I felt I didn’t deserve it if I did less in training or whatever*.*’ (Age 22–24*, *current lightweight*, *intermediate)*
Disordered eating	*AMY*: *‘I can’t really look at food in the same way anymore* … *I only did it [lightweight rowing] competitively for one year and it just affected me so much*. *It spiralled when I was racing competitive*, *I felt self-conscious*. *It was a yo-yo affect like going from one extreme to another of dieting and binging after racing*. *I wish I could take back a few years*, *just go back to how I was previously because there was no issue whatsoever*.*’ (Age 19–21*, former lightweight*, *elite international)* *MIA*: *‘I think a lot of people with different opinions give you strange advice which doesn’t help when you’re 18*. *I didn’t really have anyone to talk to*, *I was feeling I was getting an eating disorder*.*’ (Age 19–21*, *former lightweight*, *intermediate)*
Negative body image	*PHILL*: *‘I think it’s worse for men* … *you’re meant to have a certain manly body type and in summer weight it is the complete opposite… I felt I should feel body negative’ (Age 19–21*, former lightweight*, *elite international)* *MIA*: *‘I was never conscious about my weight before I started doing it [lightweight rowing]*, *I’m quite slim anyway and I’ve never really been able to put weight on*. *Now I just have weird things in my head like If I’m hungry I’m like yeah that’s good… I want to eat good clean food because it is almost drilled into you*, *you just want to be the skinny girl*.*’ (Age 19–21*, *former lightweight*, *intermediate)*
**Physical implications of RED-S**	
Disrupted sleep and fatigue	*PAUL*: *‘I couldn’t get to sleep because I was so hungry* … *I would wake up a couple of times I would be so hungry I would have to have a cereal bar or something which I shouldn’t really have*.*’ (Age 19–21*, former lightweight*, *elite international)* *PIP*: *‘I was definitely overtired*. *I would sleep whenever I could*. *I did have quite a good routine generally because of rowing but sometimes if I was really hungry from dieting and stressed about that and exams I wouldn’t sleep as well*, *and I would wake up in the night hungry*.*’ (Age 22–24*, *current lightweight*, *elite national)*
Decreased performance and impaired recovery	*ANG*: *‘I tended to yo-yo like 85% of the time with dieting which didn’t help my training performance as I would just have like no energy during the sessions and my coach would comment on how awful I looked which I know was due to how little I was eating*.*’ (Age 22–24*, former lightweight*, *elite international)* *PHILL*: *‘I had to adapt my own training programme because I couldn’t train enough*, *well I couldn’t eat enough because of dieting to support the training…so we have multiple days of 18k on the erg and I was having to cut that to 12k because I couldn’t finish it without going light-headed and really struggling… I would be really tired all through the session and then I’d be tired for work…I’d be struggling because I was never recovered*.*’ (Age 22–24*, *current lightweight*, *intermediate)*
Bowel disruption	MEG: ‘Yes, I had a dodgy stomach. I cut out fibre and that made it worse… I got bloated quite a lot.’ (Age 19–21, current lightweight, elite national) *ANG*: *I took so much senokot which is a laxative to lose the weight*. *But I probably would have a doggy stomach if I didn’t have so much laxatives because I completely cut out fibre when I was gut cleansing*.*’ (Age 22–24*, former lightweight*, *elite international)*
Menstrual dysfunction	*MIA*: *I had amenorrhea for like 4 or 5 months*.*’ (Age 19–21*, *former lightweight*, *elite national*,*)* *TAL*: *‘My periods stopped a month into starting rowing in first year*, *I also wasn’t getting enough sleep*. *I didn’t really take notice at first but it didn’t come back for 3 years*, *I have been going to the GP but they didn’t help and just said maybe I had PCOS and needed to eat more and they put me on the pill which I know now was not the right thing to do*. *I think I was more aware of the real problem than the doctor*.*’ (Age 22–24*, *former lightweight*, *elite international)*
Weakened immune system	*BOB*: *‘I had glandular fever and got a few nasty colds* …*very hard to shake off*.*’ (Age 31–33*, *former lightweight*, *elite international)* *PAUL*:*’ I was more susceptible to picking up colds when I was lighter* … *It took a long time to recover from these and I would-be put-on antibiotics which obviously affects your ability to train*. *I would say I don’t pick up as many colds now not being a lightweight’ (Age 19–21*, former lightweight*, *elite international)*
Musculoskeletal pain and injuries	*BOB*: *‘Over the years*, *I’ve got some bulging discs in my lower back*, *I had knee surgery* … *I basically injured my shoulders from wear and tear and had injections… …lower back flaring up quite a lot so had epidurals for my back… yeah you get niggles all the time’ (Age 31–33*, *former lightweight*, *elite international)* *MIA*: *‘I got a bulging disc in my back; it was because I was so stripped as a lightweight*, *I wasn’t able to support myself with my muscles… I still have ongoing problems now with my spine because of this… I always got niggles*, *I was often very ill and took ages to recover’ (Age 19–21*, *former lightweight*, *elite national)*

#### Low mood and poor emotional regulation

Many participants described experiencing low mood and poor emotional regulation. They felt that hunger and dieting had a negative impact on their mood and ability to regulate emotions.

*MIA*: *‘I remember I had a driving lesson and I just cried because I was just so flat and really down and I can now look back on it now as something chemically not right because of not eating… I had cake once because it was my birthday and my mood just changed drastically*.*’*

*(Age 19–21*, *former lightweight*, *elite national)*

### Reduced social interaction and difficulty maintaining relationships

Ten participants believed lightweight rowing resulted in reduced social interaction and difficulty maintaining relationships with friends, colleagues, family and partners. Many avoided eating out as they could not ‘afford’ the calorie consumption.

*PIP*: *‘I used to stay late a lot on campus*, *just so I could eat my food by myself*. *I also would say I can’t come out to certain social’s because I either needed to do an extra cardio or I couldn’t eat anything there*.*’*

*(Age 22–24*, *current lightweight*, *elite national)*

#### Poor concentration

Participants described experiencing poor concentration attributed to hunger and the stress of maintaining a light weight. This negatively impacting their academic and employment productivity.

*PIP*: *‘It probably shifts your priorities and to what extent you’re able to focus… you can’t just finish the sport*, *and then compartmentalise because you’ve got to think about what you’re eating throughout the day*.*’*

*(Age 22–24*, *current lightweight*, *elite national)*

#### Guilt and anxiety around food

All participants believed the pressure of making weight negatively impacted their relationship with food. Participants experienced guilt and anxiety around food. All six retired participants expressed their long-term relationship with food was damaged.

*MARY*: *‘A guilt thing maybe* … *I felt like if I ate a certain amount*, *I needed to exercise it off…I’d feel like I didn’t deserve to eat*.*’*

*(Age 22–24*, *former lightweight*, *elite international)*

#### Disordered eating

Five participants described disordered eating including binge eating attributed to food cravings following extreme diet restrictions. This was most evident post weigh-in for competition.

*PHILL*: *l ‘I binged for a couple of days afterwards and I think that probably made it worse with body image and things and then I went back to being like*, *this isn’t acceptable*.*’*

*(Age 22–24*, *former lightweight*, *elite international)*

#### Negative body image

Eleven participants reported lightweight rowing resulted in a negative body image, which continued once retired. Two male participants felt societal pressure to have a masculine physique which they were unable to sustain when losing weight. Participants described experiencing anxiety and fear following retirement in anticipating how their body shape might change.

*PIP*: *‘I definitely experienced body dysmorphia; it disconnects you from what’s actually normal… I’m anxious I’m going to look a certain way*, *it’s kind of a fear that I can’t stop rowing because of how I should look*.*’*

*(Age 22–24*, *current lightweight*, *elite national)*

### Physical implications

Six themes were identified demonstrating the physical implications of RED-S in lightweight rowers, additional quotations are presented for each theme in Table 2.

#### Disrupted sleep and fatigue

Ten participants believed dieting resulted in disrupted sleep and fatigue, compromising energy levels and performance. Some experienced irregular sleeping patterns due to reduced calorie intake. Many woke up hungry, during the night or early morning and would go to sleep early in the evening, to avoid the feeling of hunger.

*MIA*: *I was so hungry I would wake up absolutely ravenous*. *I would also go to sleep really*, *really hungry and down a litre of squash and then just go straight to sleep*. *I would try and convince myself I was full*, *and I would just wake up really*, *really hungry*.*’*

*(Age 19–21*, *former lightweight*, *intermediate)*

#### Decreased performance and impaired recovery

All participants reported that weight loss tactics and dehydration resulted in decreased performance and impaired recovery. Ten athletes felt unable to recover between sessions, negatively impacting performance.

*TAL ‘It [dieting] really affected our race day performance*, *we could barely row four kilometres without people going dizzy… it was not healthy*, *and we were definitely not fuelled*.*’*

*(Age 22–24*, *former lightweight*, *elite international)*

#### Bowel disruption

Ten participants used ‘gut clearing’ to lose weight, increasing fibre intake two weeks before weighing in then drastically removing fibre, eliciting frequent bowel movements. Some participants reported this made them unwell, producing ‘painful bloating’ and ‘constipation’. Only two participants did not report bowel disruption whilst one of the two regularly used laxatives for weight loss and to reduce bowel disruption.

*MIA*: *‘In the run up to weigh in I would obviously cut fibre as well which really upset my stomach*, *I would get so bloated it would be painful*.*’*

*(Age 19–21*, *former lightweight*, *elite national)*

#### Menstrual dysfunction

Five of the eight female participants (62%) reported menstrual dysfunction (irregular menstrual cycles, primary and secondary amenorrhea). Three participants used the contraceptive pill to try and correct their disrupted cycles. One participant expressed the pill made them feel anxious, as it masked the cause of menstrual disruption.

*GRACE*: *‘… the amenorrhea started for like six months*, *seven months… I went to the doctors; they were like you need to do less exercise and eat more… you know when someone looks at you and just doesn’t understand you’*

*(Age 19–21*, *former lightweight*, *intermediate)*

#### Weakened immune system

Nine participants described having compromised health whilst competing as a lightweight rower. They reported having experienced a range of health complaints (including recurrent colds and infectious mononucleosis) and felt that they had a weakened immune system.

*ANG*: *… ‘I was just really unhealthy*… *I definitely had a few more colds that were harder to shift and wasn’t able to eat the best food to help myself recover … ‘*

*(Age 22–24*, former lightweight*, *elite international)*

#### Musculoskeletal pain and injuries

Eleven rowers experienced musculoskeletal pain and injuries from lightweight rowing, ten experienced recurrent injuries. Common injuries included rib stress fractures, intervertebral disc extrusions, generalised joint pain, chronic wrist and ankle injuries. One retired participant described their injuries still impacting daily life, limiting participation in exercise.

*AMY*: *‘… pretty much 90% of my days have been pain*, *it did turn out that I actually had fractured it*, *I raced with a fractured rib* … *It’s not fractured now but I have osteopenia*.*’*

*(Age 19–21*, former lightweight*, *elite international)*

## Discussion

In this study, lightweight rowers did not meet their energy needs due to high energy expenditure and restricted calorie intake, which negatively impacted their psychosocial and physical health. To our knowledge, this is the first study to explore experiences of RED-S from the perspective of lightweight rowers. These findings are supported by previous research highlighting a need for effective strategies to prevent and manage RED-S whilst improving education and support for athletes.

Energy intake must align with energy expenditure to maintain adequate physiological function, growth and repair of tissue, and to prevent illness [10]. Lightweight rowers in this study used weight loss tactics and experienced a ‘yo-yo’ effect of weight fluctuation. Weight-class sports and leanness sports are associated with an increased risk of athletes using extreme weight-loss tactics and experiencing rapid weight fluctuation, which can be detrimental to health and performance [11]. Individuals in this study felt lightweight rowing negatively affected their relationship with food, and this relationship with food often persisted once retired. Daily calorie counting can increase the risk of developing disordered eating [12]. Disordered eating among lightweight rowers is not a new phenomenon; a higher prevalence of disordered eating has been reported among collegiate lightweight rowers compared with heavyweight rowers [13]. Furthermore, of 103 elite heavyweight and lightweight rowers, the lightweights were more likely to have disordered eating, depression, confusion, and tension [14]. Disordered eating can negatively impact upon health; adversely affecting gastrointestinal, endocrine, hematologic, neurologic, bone metabolism, pulmonary and cardiac function [15], which aligns with our study findings. A qualitative study in endurance runners found that athletes’ experiences of RED-S were similar to the physical and psychological experiences expressed by lightweight rowers in our study [16]. In contrast, comparison of findings between studies suggests that the mechanisms influencing the onset of RED-S may in some cases, be sport-specific. Therefore, RED-S prevention strategies may benefit from being sport-specific and addressing specific challenges faced within a sport. Our findings emphasise the importance of awareness and education of RED-S and the associated serious health consequences.

Lightweight rowers described negative implications on their social life and relationships due to rowing, but accepted this as ‘just a sacrifice you have to make.’ Such beliefs may reduce athletes’ communication with coaches and the sports medicine team about psychosocial health, inhibiting adequate management. Aerobic exercise can increase cognitive function including memory and executive functions [17], however, nutrient deficiencies are associated with reduced productivity and depressive symptoms in school-aged children [18], aligning with the lightweight rowers in this study who experienced difficulty concentrating, low energy levels and mood swings. Additionally, negative experiences as a lightweight rower may affect physical activity levels across the lifespan. Ten participants reported a negative relationship with exercise due to lightweight rowing and excessive training which made them less inclined to participate in exercise after retirement. A survey that asked 293 individuals about past sport participation, revealed those who had negative memories or experiences during sport were more likely to cease participation in sport or physical activity in later life due to such experiences [19]. Maintaining a physically active lifestyle across the lifespan plays an important role in sustaining adequate physical and mental health, highlighting the importance of promoting positive experiences in sport. If lightweight rowers have negative experiences due to dieting, excessive exercise, and weight loss, it may make them less inclined to continue with physical activity once retired from lightweight rowing.

Lightweight rowers in this study described difficulties sleeping, resulting in physical and psychological consequences. Sleep has numerous vital physical and psychological functions for humans, and is one of the most crucial contributions to successful athletic performance [20]. Sleep can increase vigour, learning ability, and mood [20]. Insufficient sleep can negatively impact athlete’s performances due to fatigue, reducing metabolism, endocrine function, cognition, immunity, pain perception and inflammation [20]. Thus, impaired sleep in lightweight rowers may contribute to other findings including recurrent injury and illness, fatigue, low mood, poor concentration, and reduced performance.

This study had a number of limitations and strengths. The interviewer’s personal experience with RED-S enabled a strong rapport with participants, facilitating in-depth responses. However, the personal experiences of the interviewer may have biased interpretation of findings. Participants provided thorough responses, however a limitation of this study is that some information may not be disclosed due to the sensitivity and stigma attached to RED-S. By interviewing lightweight rowers of a range of competition standards and both sexes, the generalisability of findings is increased, but not to a wider population outside the UK. Additionally, our study was not designed to explore differences in RED-S experiences between male and female athletes, or between athletes competing at different competition levels. This could be a fruitful area for future research. Conducting telephone interviews rather than face-to-face interviews reduces response bias, whilst increasing validity with potentially resulting in more honest answers as there is no fear of judgment [21]. Using Twitter for recruitment relied upon participants having social media; potentially leading to selection bias and reducing generalisability. Nevertheless, social media provides a practical and efficient way of recruiting. Former lightweight rowers reflected upon past experiences, potentially resulting in recall bias but providing important insights into the long-term impact of RED-S.

## Conclusion

Restricting calorie intake whilst increasing energy expenditure to ‘make weight’ had significant physical and psychosocial implications for lightweight rowers. The physical impacts of RED-S included disrupted sleep, fatigue, decreased performance, impaired recovery, bowel disruption, menstrual dysfunction, weakened immune system, musculoskeletal pain, and injuries. The psychosocial impacts included low mood, poor emotional regulation, reduced social interaction, difficulty maintaining relationships, poor concentration, guilt and anxiety around food, disordered eating, and negative body image. These findings highlight the importance of effective strategies to prevent and manage RED-S in lightweight rowers.
